# Supplementary material for: Comparative Characterization of Volatile Compounds of Ningxiang Pig, Duroc and Their Crosses (Duroc × Ningxiang) by Using SPME-GC-MS
Source: Foods. 2023 Mar 2;12(5):1059. doi: 10.3390/foods12051059 (PMC10001212; doi:10.3390/foods12051059)
Supplement: Supplementary file 1 [file foods-12-01059-s001.zip › foods-2185913-supplementary.pdf]

Supplementary material

Table S1. Determination of flavor compounds in three different pork by GC-MS.

Category	Material	Populations		
		NX（%）	DC（%）	DN（%）
Alcohol	Pentan-1-ol	—	0.52	0.14
	1-Pentadecanol	1.69	0.1	1.65
	6-Pentadecen-1-ol,(6Z)-	—	0.36	0.14
	1-Eicosanol	0.55	0.54	0.33
	Decan-1-ol	0.08	0.14	0.1
	1-Nonanol	0.1	0.1	0.12
	1-Nonen-3-ol	0.05	—	0.08
	1-Hexadecanol	1.87	2.59	3.02
	1-Heptadecanol	0.19	0.18	0.21
	1-Tetradecanol	—	0.79	—
	Oct-1-en-3-ol	1.25	1.14	1.42
	2,4-Dimethylcyclohexan-1-ol	0.25	0.43	0.26
	7-Tetradecen-1-ol	0.1	0.25	0.32
	Trans-2-Octen-1-ol	—	0.22	0.14
	Cis-7-tetradecenol	0.16	—	0.27
	1-Dodecanol	1.5	2.05	2.12
	Heptan-1-ol	0.64	1.1	0.74
	1-Hexanol	0.09	0.32	0.14
	1-Octanol	1.37	1.83	1.57
Total		9.89	12.66	12.77
Aldehyde	Trans-2-undecenal	0.22	0.21	0.26
	(E,E)-2,4-Nonadienaldehyde	0.17	0.46	0.12
	Trans-2-trans-4-Octadienal	—	0.08	—
	(e,z)-2,6-Dodecadien-1-al	0.07	—	—
	(4E)-4-Undecenal	0.12	0.45	0.18
	(9Z)-Octadeca-9,17-Dienal	0.13	0.11	0.16
	Trans-2-Heptenal	0.3	0.93	0.24
	(E,E)-2,4-Heptadienal	—	0.08	—
	(E,E)-2,4-Dodecadien-1-al	0.07	0.22	0.07
	Cis-13-Octadecenal	0.12	—	—
	(Z)-14-Methyl-8-Hexadecen-1-al	0.07	—	0.08
	Cis-4-Decenal	0.25	0.23	0.27
	(Z)-7-Hexadecenal	—	1.17	0.45
	(Z)-Hexadec-9-enal	3.49	1.78	4.54
	(Z)-9-tetradecenal	0.02	0.31	0.02
	(Z)-Octadec-9-enal	—	0.34	—
	(E)-2-Hexadecenal	0.03	0.03	—
	2,4-Decadienal	0.48	—	0.49
	(E,E)-2,4-Dodecadien-1-al	—	—	0.03
	2,4-Undecadienal	—	0.18	0.08
	2-Butyl-2-Octenal	0.03	0.2	0.07
	(E)-2-Tridecen-1-al	0.49	0.25	0.47
	2-Undecena	6.77	7.84	6.98
	2-Ethyl-2-Hexenal	—	0.09	—
	3,5-Dimethylbenzaldehyde	0.1	—	0.07
	4-Pentylbenzaldehyde	0.63	0.81	0.7
	4-Ethylbenzaldehyde	—	0.3	—
	Benzaldehyde	1.47	2.33	0.98
	Phenylacetaldehyde	—	0.08	—
	(2E,4E)-Deca-2,4-Dienal	3.35	4.09	2.23
	Trans-2-Dodecenal	0.09	0.18	0.2
	(2E)-2-Octenal	0.73	1.21	0.61

	Trans,Trans-2,4-Heptadienal	0.02	—	—
	(2E)-2-Decenal	0.47	3.47	0.12
	(2E)-2-Nonenal	0.92	1.83	0.8
	Heptanal	0.68	1.45	0.84
	Decanal	0.31	0.46	0.37
	(2-Trans,6-Trans)-Farnesal	0.04	0.04	0.02
	5,9,13-Trimethyl-4,8,12-Tetradecatrienal	0.07	0.05	0.05
	Nonanal	4.95	5.96	5.4
	Octadecanal	3.24	1.05	3.24
	Dodecanal	0.77	1.08	1.04
	Hexadecanal	10.08	—	8.54
	Hexadeca-7c,10c-Dienal	0.09	0.18	0.11
	Tridecanal	1.34	2.36	2.75
	Tetradecanal	24.27	11.83	20.29
	Pentadecanal	—	4.32	—
	Undecanal	0.33	0.47	0.41
	Z-7-Tetradecenal	0.47	—	0.42
	Pentanal	—	0.36	—
	Hexanal	1.92	3.38	2
	Octanal	2.49	3.33	2.81
Total		71.66	65.58	68.51
Ketone	1-Cyclopentylprop-2-yn-1-one	—	0.08	0.11
	2-Heptanone	—	0.07	—
	Nonadecan-2-one	0.17	—	—
	2-Tetradecanone	—	0.17	0.06
	Nona-3,5-dien-2-one	0.06	—	—
	6-Methyl-2-Heptanone	—	0.21	—
	6-Undecyloxan-2-one	—	—	0.03
Total		0.23	0.53	0.2
Alkane	2-Ethyl-1,6-Dioxaspiro[4.4]nonane	0.05	0.05	0.09
	3-Bromodecane	—	0.09	—
	9-Methylbicyclo[3.3.1]nonane	—	0.06	0.02
	Cyclohexadecane	0.42	0.22	0.39
	Cyclotetradecane	2.02	0.75	2.58
	Dodecane	0.04	—	0.03
	Pentadecane	0.11	0.46	0.11
	Hexadecane	0.54	0.43	0.27
	Tridecane	0.12	0.18	0.13
	Tetradecane	0.11	—	0.09
Total		3.41	2.24	3.71
Alkene	Cyclododecene	—	—	0.09
	1,13-Tetradecadiene	0.04	—	0.07
	1-Pentadecene	—	—	0.04
	3-[(E)-3-methylbut-1-enyl]Cyclohexene	0.06	0.06	0.05
	(3E)-3-Ethyl-2-methyl-1,3-Hexadiene	0.48	0.87	0.47
	4,6-Decadiene	0.04	0.11	0.04
	5-Hexyl-3,3-Dimethylcyclopentene	—	0.19	0.07
	(E)-Undec-5-ene	0.03	—	0.03
	Trans-Octadec-9-ene	—	0.06	—
	(Z)-2-Decen-1-al	4.05	2.81	4.24
Total		4.7	4.1	5.1
Ester	γ-Palmitolactone	0.03	—	0.05
	Methyl N,N-Diethylcarbamodithioate	0.16	0.12	0.14
	γ-Dodecanolactone	—	0.09	—
	Formic acid,Octylester	—	0.08	0.02
	Diisobutyl phthalate	—	0.16	—
	Dodecyl formate	0.51	—	0.47
Total		0.7	0.45	0.68

Acid	2-Methylamino-5-(trifluoromethyl)-1,3,4-Thiadiazole	0.17	—	—
	Pelargonic acid	—	—	0.1
	Tetradecanoic acid	0.41	0.28	0.33
	Cis-Vaccenic acid	0.13	—	0.13
	Stearic acid	0.07	—	0.04
	Dodecanoic acid	0.1	—	—
	Pentadecanoic acid	0.47	—	0.41
	Palmitic acid	2.26	1.23	1.92
Total		3.61	1.51	2.93
Furfuran	2-Pent-2-Enylfuran	—	—	0.1
	2-Heptylfuran	0.17	0.28	0.25
	2-Pentylfuran	1.5	2.23	1.73
Total		1.67	2.51	2.08

Table S2. Relative contents of eight substances in NX.

Category	Count	Peak area	Relative content (%)
Alcohol	15	7.17×10^{6}	9.89
Aldehyde	41	5.20×10^{7}	71.66
Ketone	2	1.67×10^{5}	0.23
Alkane	8	2.47×10^{6}	3.41
Alkene	6	3.41×10^{6}	4.7
Ester	3	5.07×10^{5}	0.7
Acid	7	2.62×10^{6}	3.61
Furfuran	2	1.21×10^{6}	1.67
Other	4	2.99×10^{6}	4.13
Total	88	7.25×10^{7}	100

Table S3. Relative contents of eight substances in DC.

Category	Count	Peak area	Relative content (%)
Alcohol	17	1.06×10^{7}	12.66
Aldehyde	44	5.47×10^{7}	65.58
Ketone	4	4.42×10^{7}	0.53
Alkane	8	1.87×10^{6}	2.24
Alkene	6	3.42×10^{6}	4.1
Ester	4	3.75×10^{5}	0.45
Acid	2	1.26×10^{6}	1.51
Furfuran	2	2.09×10^{6}	2.51
Other	3	8.68×10^{6}	10.42
Total	90	8.33×10^{7}	100

Table S4. Relative contents of eight substances in DN.

Category	Count	Peak area	Relative content (%)
Alcohol	18	1.15×10^{7}	12.77
Aldehyde	40	6.18×10^{7}	68.51
Ketone	3	1.81×10^{5}	0.2
Alkane	9	3.35×10^{6}	3.71
Alkene	9	4.60×10^{6}	5.1
Ester	4	6.14×10^{5}	0.68
Acid	6	2.64×10^{6}	2.93
Furfuran	3	1.88×10^{6}	2.08
Other	3	3.63×10^{6}	4.02
Total	95	9.03×10^{7}	100
